# Biomedical applications of snake venom: from basic science to autoimmunity and rheumatology

**DOI:** 10.1016/j.jtauto.2020.100076

**Published:** 2020-12-14

**Authors:** Carlos A. Cañas, Santiago Castaño-Valencia, Fernando Castro-Herrera, Felipe Cañas, Gabriel J. Tobón

**Affiliations:** a GIRAT: Grupo de Investigación en Reumatología, Autoimunidad y Medicina Traslacional, Fundación Valle Del Lili and Universidad Icesi, Cali, Colombia; b Fundación Valle Del Lili, Rheumatology Unit, Cra 98 No. 18 - 49, Cali, 760032, Colombia; c Department of Physiological Sciences, Department of Health Sciences, Universidad Del Valle, Cali, Colombia; d Department of Cardiology, Clínica Medellín, Medellín, Colombia

**Keywords:** Snake venoms, Autoimmunity, Translational medicine

## Abstract

Snake venoms have components with diverse biological actions that are extensively studied to identify elements that may be useful in biomedical sciences. In the field of autoimmunity and rheumatology, various findings useful for the study of diseases and potential drug development have been reported. The study of disintegrins, proteins that block the action of integrins, has been useful for the development of antiplatelet agents and principles for the development of immunosuppressants and antineoplastics. Several proteins in snake venoms act on the coagulation cascade, activating factors that have allowed the development of tests for the study of coagulation, including Russell’s viper venom time, which is useful in the diagnosis of antiphospholipid syndrome. Neurotoxins with either pre- or postsynaptic effects have been used to study neurogenic synapses and neuromuscular plaques and the development of analgesics, muscle relaxants and drugs for neurodegenerative diseases. Various components act by inhibiting cells and proteins of the immune system, which will allow the development of anti-inflammatory and immunosuppressive drugs. This review summarizes the usefulness of the components of snake venoms in the fields of autoimmunity and rheumatology, which can serve as a basis for diverse translational research.

## Highlights

• Snake venoms possess multiple biological functions useful in translational research.
• Snake venoms have various biological effects on the immune system.
• Many answers about autoimmunity can be solved based on the study of snake venoms.

## 1 Introduction

Snake venom contains a complex mixture of proteins with different biological effects, whose primary functions are to immobilize prey, kill it and, in the case of vipers, start the digestive process. These properties are also useful when defending against predators. Human beings become a part of this interaction when they are bitten and experience envenomation. Many of the effects of venoms are quite noticeable and diverse at the physiological and clinical levels, which has led to their study in different areas [1]. This knowledge has been useful for the development of biomedical applications such as the study of the pathogenesis of various diseases, the design of diagnostic tests, and the development of drugs. Some of the effects of venoms may have an impact on different physiological aspects, mainly related to the immune system, not visible during envenomation.

Many of the components of venom have been isolated, characterized, and assessed for their biological actions. Their medical utility was rapidly discovered [2]. Relevant biological actions studied within the scope of biomedical use of venoms include the following: the effects of disintegrins on the adhesion of cells, the effects of L-amino acid oxidases (LAAOs) on cell dynamics and promotion of apoptosis, platelet function and coagulation factors, the effects of α neurotoxins (belonging to the three-finger toxin (TFT) family) and β neurotoxins, hypotensive effects, potassium channel blockade and the biological effects of nerve growth factors (NGFs), the effects of myotoxins, cardiotoxins, and TFTs with other types of actions different from their effects as α-neurotoxins and effects on the immune system, among others.

The present article describes the most relevant aspects of this form of translational medicine that have been proven and will prove to be useful in the field of autoimmunity and rheumatology. Some knowledge in the field of oncology and hematology is included, which is a useful complement.

Effects on cell adhesion: snake venom disintegrins as basis for the study and development of treatments for inflammation, thrombosis and neoplastic processes.

Multicellular organisms require the integration of cells with each other and with the extracellular matrix through proper adhesion, a process that has incorporated different types of molecules throughout evolution [3]. These adhesion molecules also play a role in intercellular communication, which, in turn, contributes to the regulation of proliferation, survival, and differentiation. Furthermore, the regulation of cell orientation and shape, as well as participation in directional cell movement, is achieved through the organization of its cytoskeleton [4]. Cell adhesion molecules are grouped into four important families: cadherins, some members of the immunoglobulin superfamily, selectins, and integrins. In cell–cell interactions, there is a receptor on the surface of a cell, while its specific ligand is on the surface of the adjacent cell. Some receptor-ligand interactions are homotypic (between similar molecules), while others are heterotypic (between different types of molecules). Homotypic adhesion is present in cadherins and some immunoglobulins. Heterotypic adhesion occurs between integrins and immunoglobulins and between selectins and glycoproteins [5].

With the exception of mature erythrocytes, all cell types have one or more integrins expressed on their surface. In mammals, approximately 20 different integrins have been found. In some cell types, the membrane-expressed receptors are mostly integrins, as in the case of platelets, which have approximately 80,000 copies of the αIIβ3 receptor (GpIIbIIIa) on their cell surface [6].

**Fig. 1:**
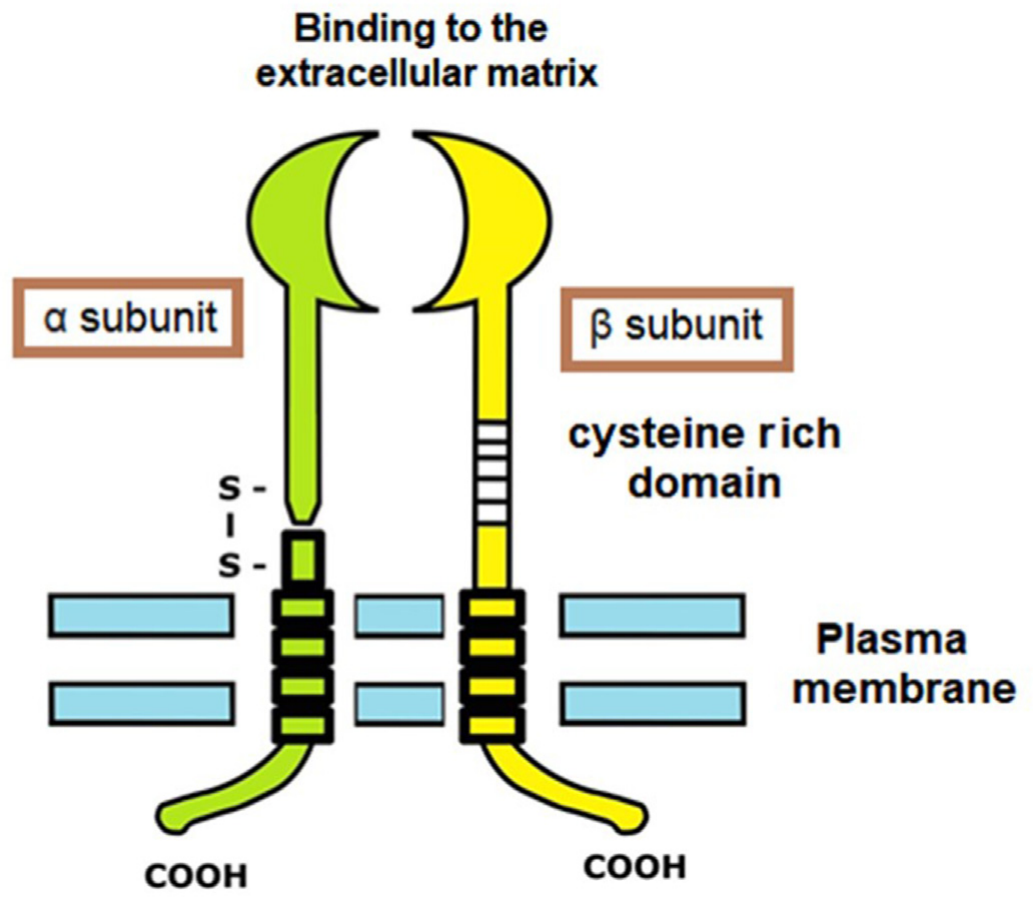
Integrins are transmembrane glycoproteins consisting of two protein chains or subunits, one α chain and one β chain; the α subunit is made up of two chains linked by a disulfide bridge, and the extracellular domain of the β subunit contains a region rich in cysteine repeats.

Integrins are transmembrane glycoproteins consisting of two protein chains or subunits, one α chain and one β chain; the α subunit is made up of two chains linked by a disulfide bridge, and the extracellular domain of the β subunit contains a region rich in cysteine repeats (Fig. 1). These molecules can be classified based on how they bind to the extracellular matrix or to other cells:

- With the extracellular matrix, they bind to fibronectins, laminins, or collagens. These junctions can be dependent on three specific amino acid motifs (e.g., RGD: arginine-glycine-aspartic acid or KGD: lysine-glycine-aspartic acid).

- With other cells, they bind through ligands of the immunoglobulin family (ICAMs, VCAM-1, MAdCAM-1) or with a cadherin (αEβ7-E-cadherin).

The β1 integrin family comprises twelve heterodimers that share CD29 as a β1 subunit. However, they are different molecules and have different properties and characteristics relevant to the immune response, especially that of lymphocytes. The β2 integrin family shares the β2 subunit, also called CD18, which consists of four heterodimers with different α subunits (also called CD11): CD11a CD18 (LFA-1), CD11b CD18 (Mac-1, CR3), CD11c CD18 (p150.95, CR4) and CD11d CD18, which are also relevant in lymphocyte and complement receptor roles. The β3 integrin family comprises two αIIbβ3 receptors (glycoprotein IIb/IIIa, CD41/CD61) and αVβ3 (CD51/CD61), which share the same β3 subunit (CD61) and participate in the platelet adhesion function. Integrins β4, β5, β6 and β8 only have one form and are expressed in various epithelial cells. Two β7 integrins have been described that are expressed in various tissues but primarily in the spleen and lymph nodes [7].

The disintegrins in snake venom act by stimulating and inhibiting integrins, altering their natural adhesion to molecules of the immunoglobulin family or to extracellular matrix structures. Their biological effects are derived from this interaction. Snake venom disintegrins have played an important role in the study of integrins, such as their location and function [8,9].

### 1.1 Disintegrins and inflammation

Disintegrins are strong candidates to be exploited in the development of anti-inflammatory and antiangiogenic therapies for chronic inflammatory processes. Immune cell movement, intercellular adhesion and binding to microenvironments are mediated by integrins, which may be antagonized by snake venom disintegrin [10]. In animal models of inflammation, alternagin-C, a disintegrin obtained from the venom of *Bothrops alternatus*, can modulate cellular behaviors such as adhesion, migration and proliferation, as well as the production of various growth factors via α2β1 integrin, important processes during inflammation and angiogenesis, which, although they appear as distinct events, act concomitantly in several chronic inflammatory diseases [[11], [12], [13]]. Similar effects have been reported with jararhagin, from *B. jararaca* snake venom in a murine sponge model [14,15]. Jararhagin also regulated the release of proinflammatory cytokines (IL-1beta, IL-6 and TNF-alpha) from murine peritoneal adherent cells after treatment with LPS (lipopolysaccharide) [16].

Rhodostomin from *Calloselasma rhodostoma* snake venom interacts with αvβ3 integrin on monocytes/macrophages, leading to interference with the activation of phagocytes triggered by LPS, suggesting a protective function of this disintegrin in LPS-induced endotoxemia due to its anti-inflammatory activity in vivo [17].

Trimucrin from *Trimeresurus mucrosquamatus* suppresses LPS-induced activation of phagocytes primarily through blockade of NF-κB and MAPK activation [18].

### 1.2 Disintegrins and thrombosis

When a wound is bleeding, a process called “primary hemostasis” begins, with platelets as the main protagonists; these cells can form a thrombus in a few seconds through their adhesion to proteins of the extracellular matrix, activation accompanied by the release of granules and aggregation between them. Subsequently, “secondary hemostasis” begins with the intervention of coagulation factors that result in fibrin formation. Both events are closely related and self-regulated.

Platelets adhere to vascular subendothelial collagen through receptors such as α2β1 integrin (glycoprotein Gp IaIIa), the FcγRIIA receptor of the crystallizable fragment of immunoglobulin G, GpVI and through GpIb/IX, through the intermediate stabilizing molecule von Willebrand factor. Subsequently, platelets aggregate as a result of the binding of fibrinogen to its receptor, αIIbβ3 integrin (Gp IIb/IIIa or CD41/CD61) [19].

Snake venoms affect blood coagulation and platelet function in diverse ways. Some venom components inhibit platelet function, while other components induce platelet aggregation. Among platelet aggregation inhibitors, disintegrins have been recognized as unique and potentially valuable tools for examining cell-matrix and cell-cell interactions and for the development of antithrombotic and antiangiogenic agents [20].

The inhibition of RGD-dependent integrins is an important objective of pharmacological research for many diseases. In thromboembolic disorders, the main objective is to block platelet αIIβ3 integrin (Gp IIb/IIIa), which binds to fibrinogen to form thrombi. The disintegrin structure has been used as a template for the design of high-affinity compounds that bind to fibrinogen. This strategy resulted in the introduction of two drugs, eptifibatide and tirofiban. Eptifibatide was modeled from the active site of the barbourin toxin of *Sistrurus m. barbouri* [21], whereas tirofiban was designed based on echostatin [22], a synthetic compound that mimics RGD [23]. Both drugs have been approved for the treatment of acute coronary syndrome and for the prevention of thrombotic complications in patients undergoing balloon angioplasty and stents [24,25].

Disintegrins with an effect on the GpIb complex have also been used to study Bernard-Soulier’s disease [26], a glycoprotein Ib congenital deficiency; this molecule is the receptor for von Willebrand factor. Patients with this disease show hemorrhagic phenomena.

Other toxins with effects on platelet function are bilinexin from *Agkistrodon bilineatus* [27], trigramin from *T. gramineus* [28], EMF10 from *Eristocophis macmahoni* [29], CC8 from *Cerastes cerastes*, EC5 from *Echis carinatus*, VLO5 from *Vipera lebetina obtuse* [30], accutin from *A. acutus* [31], triflavin from *T. flavoviridis* [32], rhodostomin from *C. rhodostoma* [33], bourin from *S. m. barbouri* [34] and DisBa-01 of *B. alternatus* [35].

### 1.3 Disintegrins and neoplastic processes

Integrins have been studied for their possible use as antineoplastic drugs, mainly due to their alleged inhibitory effect on neoplastic cell adhesion to the extracellular matrix, a process that would prevent the development of metastasis. Antiangiogenic and proapoptotic effects have also been reported in diverse neoplastic cell lines. *In vitro* studies have been carried out on viperistatin [36] from *V. lebetina obtusa* and its effect on melanoma cells; obtustatin [37,38] also from *V. lebetina obtusa* in melanoma; viridistatin [39] from *Crotalus viridis viridis* in six different cancer cell lines (bladder cancer, fibrosarcoma, melanoma, colorectal adenocarcinoma, breast cancer, and murine melanoma); and r-mojastin-1 [40] from *C. scutulatus scutulatus* in bladder carcinoma, human fibrosarcoma, human melanoma, and murine melanoma. Contortrostatin from *A. contortrix contortrix* has additionally been studied because of its antiangiogenic effect [[41], [42], [43]]. Salmosin [[44], [45], [46]] from *A. h. brevicaudus* has been studied because of its antiangiogenic and apoptosis-inducing effects. Bitistatin [47] from *Bitis arietans* and echistatin [48] from *E. sochureki carinatus* have, in addition to an antiangiogenic effect, other effects that alter intracellular dynamics by inhibiting cellular function.

Table 1 shows the different integrins with their alternate names, cell location and ligands (some include the motifs or triplet of amino acids related to the junction), as well as the ophidotoxins known to act on them and the snake species in which they have been reported.

**Table 1:** Integrins’ characteristics and ophidotoxins with action on them.

Integrin	MainLocation	MainLigand	Toxin	Snake species
α1β1(VLA-1, CD49a/CD29)	T lymphocytesSmooth muscleFibroblstsMonocytes	LamininsCollagen(KTS motif)(RTS motif)	Obtustatin [27,38]Viperistatin [36]JerdostatinLebestatin	*Vipera lebetina obtuseTrimeresurus jerdonii Vipera lebetina*
α2β1(VLA-2, Ia/IIa, CD49b/CD29)	T and B lymphocytesMonocytesPlateletsFibroblastsLangerhans cells	LamininsCollagen	Rhodocetin [33]Bilinexin [27]	*Calloselasma rhodostoma Agkistrodon bilineatus*
α4β1(VLA-4, CD49d/CD29)	T and B lymphocytesMonocytesMastocytesEosinophilsPlateletsFibroblastsLangerhans cells	MadCAM Trombospondin, Fibronectin VCAM-1(MLD motif)	EC5 [30] VLO5(30)	*Echis carinatus* *Vipera lebetina obtuse*
α5β1(VLA-5, CD49e/CD29)	T lymphocytesPlateletsFibroblastsMonocytesMastocytes	Fibronectin (RGD motif)(MGD motif)(WGD motif)(MLD motif)	Trigramin [28]Contortrostatin [[41], [42], [43]]EMF10 [27]CC8(30)EC5 [30]VLO5(30)	*Trimeresurus gramineus Agkistrodon contortrix contortrixEristocophis magmahoni Cerastes cerastes Echis carinatus Vipera lebetina obtuse*
α9β1(VLA-9, CD49i/CD29)	Multiple cells, especially myocytes	Collagen(MLD motif)	EC5 [30] VLO5 [30]	*Echis carinatus Vipera lebetina obtuse*
αVβ3(CD51/CD61)	Platelets	Laminin Fibronectin Vitronectin Von Willebrand factor Thrombospondin (RGD motif) (WGD motif)	Viridistatin [39] Accutin [6] Triflavin [32] Salmosin [[44], [45], [46]] Trigramin [28] Contortrostatin [[41], [42], [43]] Bitistatin [47] r-Mojastin 1 [40] CC8 [30]	*Crotalus viridis viridis* *Agkistrodon acutus* *Trimeresurus flavoviridis* *Agkistrodon h. brevicaudus* *Trimeresurus gramineus* *Agkistrodon contortrix contortrix* *Bitis arietans* *Crotalus scutulatus scutulatus* *Cerastes cerastes*
αIIbβ3 (glicoproteína IIbIIIa, CD41/CD61)	Platelets	Factor von Willebrand Fibrinogen, Fibronectin (RGD motif) (KGD motif) (MLD motif) (WGD motif)	Trigramin [28] Contortrostatin [[41], [42], [43]] Rhodostomin [33] Barbourin [21,34] DisBa-01z [34] Triflavin [32] EC5 [30] VLO5(30) CC8 [30]	*Trimeresurus gramineus* *Agkistrodon contortrix contortrix* *Calloselasma rhodostoma* *Sistrurus m. barbouri* *Bothrops alternatus* *Trimeresurus flavoviridis* *Echis carinatus* *Vipera lebetina obtusa* *Cerastes cerastes*
αvβ5	Various epithelial cells. Example: placenta	Vitronectin (RGD motif)	Trigramin [28] Contortrostatin [[41], [42], [43]]	*Trimeresurus gramineus* *Agkistrodon contortrix contortrix* *Agkistrodon contortrix contortrix*
α4β7 (LPAM-1)	Various epithelial cells. Example: spleen and lymph nodes	Fibronectin VCAM-1 (MLD motif)	EC5(30) VLO5(30)	*Echis carinatus* *Vipera lebetina obtusa*

PROAPOPTOTIC EFFECT: THE ROLE OF L-AMINO ACID OXIDASES IN THE STUDY AND DEVELOPMENT OF ANTINEOPLASTIC, IMMUNOSUPPRESSIVE AND ANTI-INFLAMMATORY DRUGS.

L-Amino acid oxidases (L-AAOs) catalyze the oxidative deamination of L-α amino acids, leading to the production of ammonium and hydrogen peroxide. The L-AAOs of snake venoms induce local alterations such as hemorrhage and edema. These molecules can have an effect on platelets, deteriorating [49] or stimulating [50] their species-dependent aggregation. *In vitro* studies have demonstrated antibacterial [51], antiviral [52] and proapoptotic effects through an oxidative stress effect [53]. The latter has been studied *in vitro* to determine its possible antineoplastic and immunosuppressant roles [54]. For instance, a selective cytotoxic effect has been described in cancer cell lines for the L-AAO from *C. vipera* venom [55], as well as that of *C. rhodostoma* [56], *C. durissus terrificus* [57] or *Ophiophagus hannah* [58], *B. atrox* [59] or *B. jararaca* [60], among others.

The toxin BjussuLAAO-II from *B. jararacussu* induces oxidative stress and DNA damage and upregulates the inflammatory cytokine genes TNF and IL6, a potential anti-inflammatory effect for exploration [61].

SNAKE VENOM PROTEASES INTERFERING WITH COAGULATION PROTEINS: UTILITY IN THE STUDY OF COAGULATION AND IN THE DEVELOPMENT OF ANTICOAGULANTS AND FIBRINOLYTICS.

Once the vascular endothelium is damaged and platelets begin to promote adhesion and aggregation, plasma proteins of the intrinsic coagulation pathway are activated and initiate the process of secondary hemostasis. The Hageman factor (XII) is cleaved and converted into activated factor XII (XIIa), which occurs through its binding to high-molecular-weight kininogens (HMWK) and to prekallikrein, which in turn is activated when transformed in kallikrein. HMWKs, prekallikrein, and factor XII form a complex linked to subendothelial collagen. Factor XIIa has a protease action; it activates other prekallikrein molecules and converts factor XI into factor XIa. In turn, factor XIa converts factor IX into factor IXa, which, together with factor VIIIa, activates factor X in a calcium, phospholipid-dependent reaction. Alternatively, both factors IX and X can activate factor VII of the extrinsic pathway of coagulation. Typically, this extrinsic pathway is activated by the conversion of factor VII to factor VIIa by the action of lipoproteins (called tissue factor), present in cell membranes, in a calcium-dependent reaction caused by cell damage. Factor VIIa can also activate factor X. This step is the point of confluence of both coagulation pathways. The final step is the conversion of prothrombin to thrombin in the presence of factor Xa, Va, calcium and phospholipids (prothrombinase complex) [62].

Thrombin has several functions as a feedback activator and regulator of the coagulation cascade. These roles include converting fibrinogen to fibrin, activating factor V, activating factor VIII, activating factor XIII (fibrin clot stabilizer protein), stimulating platelet aggregation and secretion, inducing the action of the plasminogen activator and activating the vascular endothelium in conjunction with thrombomodulin and C and S proteins, which inhibit factors Va and VIIIa, thus exerting an anticoagulant or regulatory action throughout the entire process described [63].

Fibrinogen comprises three pairs of polypeptide chains: two Aα, two Bβ and two γ. These polypeptides are bound together by 29 disulfide bonds so that the N-terminal regions of the six polypeptide chains together form a central E domain forming a bond with an α-helix structure or bridge between the two N-terminal portions of the two Aα chains. The N-termini of the Aα and Bβ chains are part of fibrinopeptides A (FPA) and B (FPB). The C-terminal regions (Aα, Bβ, and γ) form D domains on each side. Thrombin splits off both FPA and FPB to form fibrin monomers, which form polymers that are stabilized when additionally activated (by thrombin itself), factor XIII.

There must be activating mechanisms as well as inhibitors to maintain the balance of clot formation. After fibrin formation, fibrin degradation factors are activated through a system called “fibrinolytic”. Fibrin begins a proteolytic process mainly mediated by plasmin, whose precursor is plasminogen (inactive precursor of plasmin). Tissue plasminogen activator (tPA) and plasminogen activator inhibitors (PAI-1, α2-antiplasmin, and TAFI) are also involved in this system. The system is activated with the binding of plasminogen to fibrin and by the action of plasminogen activators, partly in response to the presence of thrombin. This fibrinolytic process generates so-called fibrin degradation products, including D-dimer.

Viper venoms are rich in proteases belonging to the families of serine proteases, metalloproteases, C-type lectins, disintegrins and phospholipases, which affect hemostasis.

### 1.4 Prothrombin activators

Snake venoms are important sources of prothrombin activators. Examples of venoms exerting this action are those of the taipan snake (*Oxyuranus s. scutellatus*), ecarin from *E. carinatus*, textarin from *Pseudonaja textilis* and noscarin from *Notechis scutatus* [64]. These venom components have been used in the study of hypoprothrombinemia [65] and disseminated intravascular coagulation [66], as well as in laboratory studies where prothrombin activation is required [67].

Resistance to activated protein C (APC) due to a mutation in factor V (factor V Leiden) is the most common form of hereditary thrombotic diathesis in humans. Its severity depends on the homozygous or heterozygous condition. This condition can be assessed through coagulation times involving prothrombin activators [68]. The first step is the inactivation of factor Va contained in a sample of the patient’s plasma when exposed to the APC. In the case of an individual without the mutation, the Va factor is easily inactivated by the APC. Then, noscarin is added [69]. In the wild-type condition, the coagulation time ​+ ​APC led to a prolongation of the coagulation time, usually 2.5 times or more than that in the control group (without activation with noscarin). Factor Va patients are resistant to APC, indicating they have factor V Leiden and are homozygous for this mutation. The clotting time is not influenced by noscarin, and the ratio with the control is 1. In the case of heterozygosity, the estimated ratio oscillates between the two extremes [55].

### 1.5 Factor V activators

Conversion of factor V into FVa can be achieved with a serine protease from *Daboia russelii* (RVV-V), which cleaves the single-chain glycoprotein at Arg1545. The enzyme can be used for tests where it is essential to know the integrity in the activation of factor V [70].

### 1.6 Factor X activators

RVV-X (also extracted from *D. russelii*) is an activator of factor X that has been useful in recognizing deficiencies in this factor and differentiating them from factor VII deficiency [56], as well as to identify lupus anticoagulants (Russell viper venom time; RVVT) [71].

### 1.7 Activators of coagulation C- protein

APC is a natural anticoagulant that inactivates FVa and VIIIa. PCA is activated by protac extracted from the venom of *A. contortrix contortrix*, which has been used in the study of congenital deficiencies of C-protein [72] and its resistance [73].

### 1.8 Fibrinogenolytic and fibrinolytic enzymes

The fibrinogenases of the venom from some snakes can cleave one or more of the fibrinogen chains, a property that has attracted much attention because of the possibility of developing fibrinogenolytic agents. The following are examples of fibrinogenases: afaacitin from Saharan horned viper (*C. cerastes*) [74] and atroxase from *C. atrox* [75] and from *V. lebetina* [76].

In contrast, fibrolase, the venom from *A. contortrix contortrix,* can degrade both the Aα and Bβ fibrin chains and shows potential as a thrombolytic agent [77]. The drug alfimeprase was produced as a recombinant truncated form of fibrolase [78] and has been studied as a thrombolytic agent in peripheral arterial occlusive disease [79] and for the dilution of central catheter thrombi [80].

### 1.9 Thrombin-like enzymes (SVTLEs)

Approximately 100 “thrombin-like enzymes” (SVTLEs) have been identified from 35 snake species [81]. These are serine proteases with active recognizable residues at site H57-D102-S195, which exert a similar action to that of thrombin, mainly cleaving FPA. A few venoms can cleave the FPB. Therefore, without the cleavage of both FPA and FPB, they cannot activate factor XIII, and the fibrin clots produced can easily be dissolved. This determines a serious form of consumption coagulopathy. These molecules have been isolated, for example, from venom from *B. atrox* (batroxobin, reptilase) and *A. contortrix* (ACTE) and from *C. rhodostoma* (Ancrod). They are currently under study for the development of anticoagulants in human use. Ancrod has been shown to be effective in limiting the volume of ischemic stroke in patients with acute thrombosis in cerebral arteries [82,83].

SVTLEs have also proven useful in the functional study of the coagulation cascade in various scenarios. Since SVTLEs are not inhibited by heparin, they can be used to study plasma samples containing this anticoagulant. *Reptilase* time is a simple alternative to thrombin time for the assessment of fibrinogen in samples containing heparin [84]. The presence of fibrin degradation products, hypofibrinogenemia, and defective fibrin polymerization will prolong the reptilase time. If this prolongation is lower than that of thrombin time, the presence of the PDFs is indirectly indicated [85].

### 1.10 Tissue plasminogen activator

Tissue plasminogen activators have been isolated from *Lachesis muta* [86] and from *T. stejnejeri* [87], among other species. Its mechanisms of action have proven useful for the development of thrombolytic therapies [88], as well as for laboratory studies [89].

Table 2 describes the enzymes of the most relevant snake venoms that have an impact on the coagulation cascade.

**Table 2:** Snake venoms’ enzymes with action on the coagulation cascade.

Enzyme action	Name	Snake species
Prothrombin activators	Noscarin [64] Ecarin [64] Textarin [64]	*Daboia russelii* *Echis carinatus* *Pseudonaja textilis* *Oxyuranus scutellatus*
Factor V activator	RVV-V [70]	*Daboia russelii*
Factor X activator	RVV-X [71]	*Daboia russelii*
Activators of coagulation C- protein	Protac [72]	*Agkistrodoncontortrix contortrix*
Fibrinogenases	Fibrolase [77] Afaacitin [74] Atroxase [75]	*Agkistrodon c.contortrix* *Cerastes cerastes* *Crotalus atrox*
Thrombin-like enzymes	Batroxobin [82] Reptilasa [82] Ancrod [83] Fibrinogenase from VL [76]	*Bothrops atrox* *Bothrops atrox* *Calloselasma rhodostoma* *Vipera lebetina*
Tissue plasminogen activator (TPA)	SV-TPA	*Trimeresurus stejnejeri* [87] *Lachesis muta* [86]

Effects of α and β neurotoxins: developing analgesics, muscle relaxants and medication for neurodegenerative diseases.

Most frequently, the neurotoxins found in snakes are α-neurotoxins, which bind to nicotinic acetylcholine receptors (nAChRs). Therefore, since acetylcholine (Ach) cannot bind to its receptor, it does not transmit the signal [90]. These molecules belong to the TFT family [91].

α-Neurotoxins have been used for the isolation and characterization of nAChR in motor end plates. The activation of central cholinergic pathways by nicotine and nicotinic agonists can induce an antinociceptive effect in a wide variety of snake venoms and has been used for the creation of acute pain models [92,93]. It has been determined that α-neurotoxins isolated from cobra venoms produce significant analgesia in animal models. Cobrotoxin, a short-chain α-neurotoxin, and α-cobratoxin, a long-chain α-neurotoxin isolated from *Naja naja atra*, have shown analgesic activity. Cobrotoxin is a specific ligand for α1-nAChR with significant central analgesic effects through an opioid-independent mechanism [94]. α-Cobratoxin shows high affinity for neuronal nAChR α7 and causes an opioid-independent antinociceptive effect [95]. Cobrotoxin can become a substitute for morphine, suppressing the withdrawal symptoms caused by its use. Likewise, an α-neurotoxin from the king cobra *O. hannah* has been used as a powerful analgesic agent (hannalgesin) [96]. Neurotoxins that recognize acetylcholine muscarinic receptors (mAChRs) have been isolated in venom from the green mamba (*Dendroaspis angusticeps)* [97]. These toxins have been useful in the investigation of the physiological functions of muscarinic receptor subtypes [[98], [99], [100]]. These muscarinic receptors have generated strong interest in the study of the pathophysiology of neurodegenerative diseases such as Alzheimer’s disease and Parkinson’s disease and have therapeutic potential against them [101].

In contrast, β-neurotoxins act at the presynaptic level and affect ACh release from presynaptic vesicles. These β-neurotoxins are responsible for high neurological toxicity with paralysis and, ultimately, apnea in the prey [102]. These principles are useful for the study and development of muscle relaxants. Crotoxin (*C. durissus*), β-bungarotoxin (*Bungarus multicinctus*), notexin (*N. scutatus*) [103] and taipoxin (*O. scutellatus*) [104] belong to the group of β-neurotoxins. Crotoxin has in vivo cytotoxic activity against neoplastic cells through mechanisms involving autophagy and apoptosis [105]. These molecules have been the subject of study in advanced cancer [106,107].

Table 3 shows the different types and examples of neurotoxins from snake venoms, the species from which it was characterized and its potential in biomedical science.

**Table 3:** Snake venoms’ neurotoxins and their potential in biomedical science.

Type of neurotoxin	Name	Snake species	Clinical utility
α-neurotoxin (postsynaptic)	Cobrotoxin α-cobratoxin [94]	*Naja naja*	Analgesia
	Hannalgesin [95]	*Ophiophagus hannah*	Analgesia
	α-neurotoxin with blocking effect for nicotinic receptors [97]	*Dendroaspis angusticeps*	Study of the pathophysiology and possible therapeutics for neurodegenerative diseases
β-neurotoxin (presynaptic)	Crotoxin [152]	*Crotalus durissus*	Antineoplastic development
	β-bungarotoxin [103]	*Bungarus multicinctus*	
	Notexin [103]	*Notechis scutatus*	
	Taipoxin [104]	*Oxyuranus scutellatus*	
Channel Blockers Kv1.1, Kv1.2 ​y Kv1.6	Dendrotoxin [108]	*Dendroaspis angusticeps*	Potassium channel study Study of the pathophysiology and possible therapeutics for neurodegenerative diseases

### 1.11 Effects on potassium channels: Development of treatments for demyelinating or neurodegenerative disorders

Apart from the neurotoxins described above (with their own mechanisms of action), dendrotoxin isolated from the African green mamba (*D. angusticeps*) by Alan Harvey et al*.* in 1979 [108] is a powerful blocker of the voltage-gated potassium channels Kv1.1, Kv1.2 and KV1.6. This property leads to neurotoxicity reflected in increased muscle activity, tremors, and fasciculations of the prey. Some structural analogs of dendrotoxin have led to the molecular recognition of different types of potassium channels, whose clinical utility is related to the study and possible development of treatments for demyelinating or neurodegenerative disorders [109,110].

### 1.12 Hypotensor effect: bradykinin potentiating factor and the development of angiotensin-converting enzyme inhibitors

As patients bitten by the snake *B. jararaca* developed severe hypotension, the mechanism of action was found to be based on the inhibition of the angiotensin-converting enzyme (ACEI). Initially, Sérgio Ferreira discovered a “bradykinin potentiating factor” in this venom [111]. Later, the English pharmacologist Sir John Vane discovered that this factor was a powerful ACEI. On the basis of this principle, researchers from the Squibb pharmaceutical laboratory developed captopril, a nonprotein ACEI, in 1975 [112]. Subsequently, several drugs were developed with this principle.

In 1982, H. Schweitz et al*.* purified the venom of the green mamba (*D. angusticeps*), a peptide structurally similar to other natriuretic peptides, which was called Dendroaspis natriuretic peptide [113]. This peptide drastically reduces the blood pressure of the prey and exerts a diuretic effect. Presently, these peptides are studied for the development of hypotensive and diuretic drugs.

### 1.13 Biological effects of nerve growth factors

The biological role of nerve growth factor (NGF) found in various snake venoms is still unknown. Its discovery in the venom from *A. piscivorus* was accidental, since what researchers truly sought was a source of phosphodiesterase, but instead they identified its NGF effect [114]. At a later stage, they developed an inhibitory antibody. These investigations led to Stanley Cohen and Rita Levi-Montalcini to be awarded the 1986 Nobel Prize in Physiology and Medicine. NGF is a widely distributed component, although in very small quantities, and is being studied as a possible neurorestorative agent [115].

Recently, a chondrogenic effect of NGF extracted from the Chinese cobra (*N. atra*) [116] has been described, which are interesting approaches for the possible development of osteoarthritis treatments.

### 1.14 Biological effects of myotoxins and cardiotoxins

.

## 2 Myotoxins

Myotoxin-A isolated in the venom from the rattlesnake *C. viridis viridis* [117] is a small protein (4600 ​Da) lacking enzymatic activity. Myotoxin specifically binds to the sarcoplasmic reticulum of muscles, causing a change in the permeability of calcium ions and thus leading to irreversible damage to the muscle fibers. These peptides have immediate action, causing instantaneous paralysis to prevent prey, which eventually dies by diaphragmatic palsy, from escaping. These molecules have been used in experimental animals to create models of muscular dystrophy [118].

Lys49 myotoxin from the Brazilian lancehead (*B. moojeni*) generates pain through myotubular ATP release mechanisms, which has allowed the study of new somatosensory and nociceptive pathways [119].

Possible antitumor [120] and antibacterial effects [121] have also been studied in ophidian myotoxins.

### 2.1 Cardiotoxins

In 1940, the existence of cardiotoxins was recognized in the venom from cobras such as *N. sputatrix* (spitting cobra) or *N. naja*, and their effects have been studied in experimental animals [122,123]. Cardiotoxins have direct lytic effects driven by a mechanism of action dependent on calcium and bind to glycosaminoglycans, which causes damage to the heart muscle through the formation of pores. This cytotoxic mechanism can be useful for the development of antineoplastic agents [124]. In 1982, Elazar Kochva et al*.* described sarafotoxin, a substance extracted from the venom of *Atractaspis enggadensis* with biochemical and biological analogy to endothelins that activates type A (ETA) and type B (ETB) endothelin receptors discovered at approximately the same time [125]. Safarotoxin causes coronary spasm and cardiac arrest, a characteristic that has proven useful in the study of microvascular physiology and heart dynamics [[126], [127], [128]].

### 2.2 Other biological effects of TFT other than their action as α neurotoxins

The first TFT discovered was α-bungarotoxin more than 50 years ago [129], and it has been widely used as an α7 nicotinic acetylcholine receptor marker. More than 600 TFTs have been described since then, and several biological effects have been found with biomedical potential [130]. Among the biological effects are impacts on neurological signal transduction, due to its action on voltage-gated sodium channels [131], on GABA receptors [[132], [133], [134]] or on α-adrenergic antagonists [135]; effects on coagulation by inhibiting platelet aggregation [136] or inhibition of factor X [137]; insulinotropic activity [138] and effects on sperm motility [139,140].

### 2.3 Other biological Effects of PLA2 and metaloproteases PLA2 with effect on membrane phospholipids

PLA2 catalyzes the hydrolysis of membrane phospholipids by separating glycerol and allowing the synthesis of prostaglandins and thromboxanes. PLA2 triggers a cascade of inflammatory events characterized by increased vascular permeability, edema formation, leukocyte recruitment into tissues, and release of inflammatory mediators, which can mimic, *in vitro,* a number of systemic and local inflammatory disorders that occur in humans. These studies have helped clarify the pathophysiological roles of these proteins in various inflammatory processes and have led to the development of anti-inflammatory, anti-neurodegenerative, and antineoplastic drugs [[141], [142], [143], [144]].

### 2.4 Myotoxic PLA2s

Myotoxins Lys49-PLA2 and Asp49-PLA_2_ from the snakes *Crotalus sp.* and *B. asper* are a class of PLA2 with catalytic activity in muscles [145,146]. These toxins can be used as tools in the study of skeletal muscle repair, as well as its regeneration [147].

### 2.5 Snake venom metalloproteases

SVMPs have different effects on tissues. One of the most relevant effects occurs at the level of the vessels, where it compromises the metabolism and structure of endothelial cells, as well as the basement membrane and cell adhesion molecules in a very selective way. These alterations can be generated through both known and unknown recipients. This opens up a very important field for the understanding of vascular physiology [148,149]. Regarding the myotoxic effect of SVMP, it is suspected that its muscular damage is partially mediated through the action of IL-6 and less likely through TNF-α [150,151].

EFFECTS ON THE IMMUNE SYSTEM NOT CLEARLY VISIBLE DURING ENVENOMATION BUT IMPORTANT WITHIN THE SCOPE OF THE STUDY OF THE IMMUNE SYSTEM AND AUTOIMMUNITY.

### 2.6 Crotoxin’s immunotherapeutic potential

Crotoxin, as previously discussed, is a β-neurotoxin that is the main toxic component of *C. durissus terrificus* and is capable of inducing neuromuscular paralysis and cardiorespiratory failure and potentiating the effect of FLA2 [152,153]. Ophidiotoxicosis by this venom, compared to that of other vipers, generates a lower inflammatory reaction and less pain, a fact that has prompted researchers to look for anti-inflammatory [154] and analgesic [155] elements in crotoxin. The immunogenicity of crotoxin is low, which has led researchers to consider the existence of an immunosuppressive effect [156,157].

The anti-inflammatory effect of crotoxin is evidenced in experimental models of inflammation in animals where the production of anti-inflammatory cytokines (IL-10 and IL-14) [158] is induced, as well as the inhibition of phagocytosis by macrophages [159] and neutrophils [160] and the effects on cell migration, which inhibit interactions with the endothelium [161,162]. Crotapotin, the crotoxin complex acid subunit, which lacks enzymatic or neurotoxic activity, acts as a chaperone, inhibiting the effect of FLA2, and it is in this sense that its role as an anti-inflammatory agent is considered [163].

The inhibitory effect of crotoxin on the components of antigen presentation [164] and on proinflammatory prostaglandins has also been assessed, as well as its possible role in the activation of regulatory T lymphocytes [165,166].

### 2.7 Actions on the complement cascade

The so-called cobra venom factor (CVF), isolated from *N. naja* [167], as well as L-AAO and serine protease from snakes of the genus *Bothrops* [168], have a complement-activating effect and have been used in the study of the complement cascade. CVF acts as a C3 analog able to deplete complement and is used in animal experiments to determine the role of the complement cascade in diverse pathological conditions, such as reperfusion injury [169], age-related macular degeneration [170], paroxysmal nocturnal hemoglobinuria [171] or myasthenia gravis [172].

Oxiagin extracted from *N. oxiana* venom inhibits the formation of C3 convertase by inhibiting the classic complement pathway. This finding is very important because of its potential for the development of inhibitory drugs for this action, which are key in the origin of various diseases, including autoimmune diseases [173].

## 3 Conclusions

Snake venoms evolved to immobilize and cause the death of prey and to initiate the digestive process. The typical bite of a viper occurs quickly to prevent the prey from attacking the snake, whose skull, for example, is very fragile. The venom generates sedation, analgesia, hypotension, myocardial depression, neuromuscular paralysis, hemorrhage, detachment of cells from their extracellular matrix, proteolysis and lipolysis of its tissues along with not clearly visible effects during envenomation that are important within the scope of the study of the immune system and autoimmunity. There are different venom proteins involved in these biological processes, and they would be very valuable if their properties were creatively applied in the search for therapeutic and diagnostic effects or in the study of various diseases, including autoimmune diseases. This is the case for the study of many snake venoms, in which various biomedical applications have been found.

## Funding

Part of this work has been funded by the Asociacion Colombiana de Reumatología (Convocatoria 2019) and Universidad Icesi (Convocatoria Interna de Proyectos-2020).

## Declaration of competing interest

The authors declare that they have no known competing financial interests or personal relationships that could have appeared to influence the work reported in this paper.
